# An in vivo pharmacokinetic study of metformin microparticles as an oral sustained release formulation in rabbits

**DOI:** 10.1186/s12917-021-03016-3

**Published:** 2021-09-25

**Authors:** Sihem Bouriche, Angela Alonso-García, Carlos M. Cárceles-Rodríguez, Farouk Rezgui, Emilio Fernández-Varón

**Affiliations:** 1 Laboratoire des Matériaux Organiques (LMO), Faculté de Technologie, Département de Génie des Procédés, Université de Bejaia, 06000 Bejaia, Algeria; 2 Department of Pharmacology, Faculty of Veterinary Medicine, Universidad de Murcia, Murcia, Spain; 3 Department of Pharmacology, Center for Biomedical Research (CIBM), University of Granada, 18071 Granada, Spain; 4 Instituto de Investigación Biosanitaria de Granada (ibs. GRANADA), Granada, Spain

**Keywords:** Metformin, Controlled-release, Microparticles, Pharmacokinetics, Polymeric-based formulations

## Abstract

**Background:**

Metformin hydrochloride is a biguanide derivative that has been widely used to treat type 2 diabetes in humans. In veterinary medicine, metformin has shown increasing potential for diabetes treatment in different species, such as equids, dogs, cats and rabbits. It is highly hydrophilic, with incomplete gastrointestinal absorption and very large variability in absolute bioavailability between species, ranging from 4% in equids to 60% in humans. Metformin also shows a short half-life of approximately 2 h in dogs, cats, horses and humans. The objectives of this study were to evaluate a poly (lactic acid) (PLA) metformin microparticle formulation to test in rabbits and conduct a pharmacokinetics study of intravenous (S_IV_) and oral solution (S_PO_) metformin administration and oral PLA microparticle (S_PLA_) administration to rabbits to evaluate the improvement in the metformin pharmacokinetics profile.

**Results:**

Metformin-loaded PLA microparticles were characterized by a spherical shape and high encapsulation efficiency. The results from Fourier transform infrared (FTIR) spectroscopy suggested the presence of interactions between metformin and PLA. X-Ray diffraction (XRD) analysis corroborated the results from the differential scanning calorimetry (DSC) studies, showing that metformin is present in an amorphous state within the microparticles. Physicochemical characterization suggested that PLA and metformin hydrochloride interacted within the microparticles via hydrogen bonding interactions. The pharmacokinetic study in rabbits showed sustained-release characteristics from the prepared microparticles with a delay in the time needed to reach the maximum concentration (T_max_), decreased C_max_ and bioavailability, and increased mean residence time (MRT) and half-life compared to the pure drug solution.

**Conclusions:**

Metformin-loaded PLA microparticles showed optimal and beneficial properties in terms of their physicochemical characteristics, making them suitable for use in an in vivo pharmacokinetic study. The pharmacokinetic parameters of the metformin microparticles from the in vivo study showed a shorter T_max_, longer MRT and half-life, decreased C_max_ and the prolonged/sustained release expected for metformin. However, the unexpected decrease in bioavailability of metformin from the microparticles with respect to the oral solution should be evaluated for microparticle and dose design in future works, especially before being tested in other animal species in veterinary medicine.

## Background

In veterinary medicine, diabetes is a chronic problem that affects different animal species; however, it is more frequently reported in dogs and cats [1] and rarely reported in other animal species, such as rabbits [2]. In equids, insulin dysregulation is the primary endocrine disorder in equine metabolic syndrome, affecting some equids with pituitary pars intermedia dysfunction (10–22% prevalence) [3, 4]. There have been several reports on the use of the hypoglycaemic drug metformin in clinical cases in different animal species [1, 3, 5]. However, its efficacy is controversial, mainly due to its low oral bioavailability [1, 3, 5].

Metformin hydrochloride, a galegine alkaloid biguanide derivative, is used as a first-line treatment for type 2 diabetes. In the presence of insulin, metformin hydrochloride acts by lowering the blood glucose concentration mainly through the inhibition of gastrointestinal glucose absorption and hepatic glucose production, increasing glucose uptake and insulin sensitivity in muscle tissue [6]. Metformin, a highly soluble hydrophilic drug with low permeability through biological membranes, is a Biopharmaceutics Classification System (BCS) class III drug. The low permeability of this drug results in poor drug absorption, which is the rate limiting step to attain suitable bioavailability. After oral administration to humans, metformin is incompletely absorbed from the gastrointestinal tract and shows 40–60% absolute bioavailability with a short elimination half-life (1.5–1.6 h) [7]. Incomplete absorption and low bioavailability also occur in some animal species, such as dogs, cats and horses [4, 5, 8]. To maintain an effective plasma concentration, metformin should be repeatedly taken at high doses (2.5 g/day), which causes serious gastrointestinal side effects [9]. In the case of animal species, when it is necessary to use the prescribing cascade principle, extrapolation from human doses is used due to the lack of knowledge of the pharmacokinetic parameters and, therefore, the most appropriate dosing regimens, which may cause problems in treated animals. In this context, in veterinary medicine, the special conditions for the treatment of animals make long-acting formulations powerful tools for use in animals affected by diabetes (and other medical conditions) [10–12].

Therefore, the development of polymeric controlled drug delivery systems (CDDSs) is an important strategy to overcome the above limitations. The purpose of these systems is to reduce side effects, sustain drug release and increase the drug bioavailability at a controlled rate for a sustained period of time (days, weeks or months) [13]. Therefore, the drug concentration can be maintained within the therapeutic window for a specified period of time. In this context, microparticles formulated with polymers have been extensively used to design CDDSs for a large number of therapeutic drugs [14, 15].

Poly (lactic acid) is a hydrophobic polymer that belongs to the α-hydroxy acid family. As a biodegradable and biocompatible substance, this polymer is widely used for many applications in the pharmaceutical and medical areas because its decomposition products are not toxic and are easily excreted from humans [16, 17]. PLA has been extensively used for encapsulation to enhance the bioavailability of many drugs for targeting and for sustained delivery purposes [18]. Several methods for the preparation of CDDS microparticles from PLA polymers have been reported in the literature. Because of their easy preparation and convenience to control process parameters, the solvent evaporation method (double emulsion) is mostly used on a laboratory scale for the preparation of microparticles. This method is especially suitable for the encapsulation of highly water soluble compounds with sufficient encapsulation efficiency [9, 19].

To our knowledge, there are no studies on the development of metformin microparticles using PLA as a polymer. Recently, optimization of metformin encapsulation/formulation in PLA microparticles in terms of encapsulation efficiency was investigated by using response surface design [20], showing potential to be used in some animal species affected by diseases including diabetes and metabolic syndrome, etc., where hyperglycaemia could be treated with antidiabetic drugs such as metformin. However, the in vivo pharmacokinetics profile of this developed metformin microparticle was not determined. For this purpose, the objectives of this study were as follows. 1) To develop and evaluate the physico-chemical characteristics of the metformin PLA microparticles to determine if this formulation is adequate to be tested in vivo in rabbits. 2) To conduct an in vivo pharmacokinetics study of metformin after S_IV_, S_PO_ and S_PLA_ administration to rabbits to evaluate the potential of this sustained-release formulation for use not only in rabbits but also in other small and large animal species.

## Results

### Encapsulation efficiency and characterization of PLA microparticles

Metformin HCl was encapsulated within PLA microparticles by the double emulsion solvent evaporation method. Batches were prepared under the same conditions as shown below in the Methods section. The results showed that the loading capacity of metformin was 16.96 ± 0.61% and the encapsulation efficiency was 76 ± 2.72%. The high observed encapsulation efficiency may be due to hydrogen bonding between PLA and metformin that may occur during the microparticle formation process, which allows high entrapment of the drug in the polymer matrix.

**Fig. 1:**
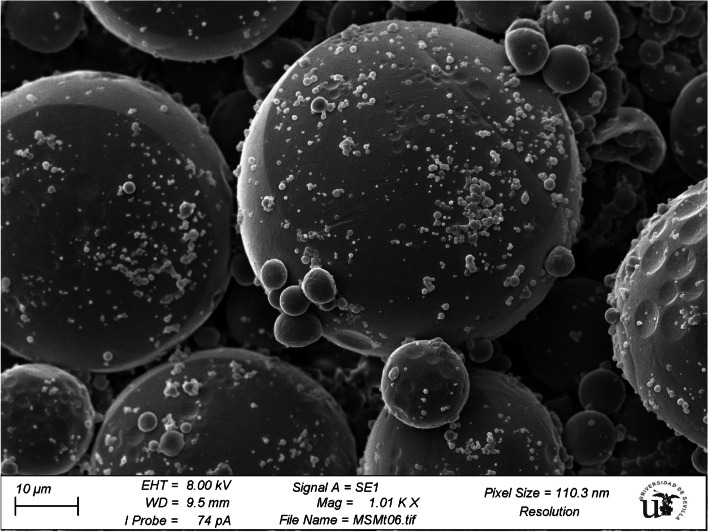
Scanning electron microscopy (SEM) images of the surfaces of the investigated metformin-loaded PLA microparticles after lyophilization. The SEM micrograph shows the large distribution of microparticle sizes, ranging from 1 to 55 µm (1010 × magnification, scale bar = 10 μm). PLA: Poly (lactic acid)

The obtained microparticles were analysed by scanning electron microscopy (SEM). An SEM micrograph of PLA-encapsulated metformin is shown in Fig. 1. The microparticles had an almost spherical shape with smooth surfaces and particles adhered to the surface. In addition to the surface morphology characteristics, it is interesting to note that the size of microparticles varies, ranging from ~ 1 to 55 µm. The SEM images also suggested that metformin was dissolved in the microparticles since no individualized drug crystals were observed.

### Fourier transform infrared (FTIR) spectroscopy

**Fig. 2:**
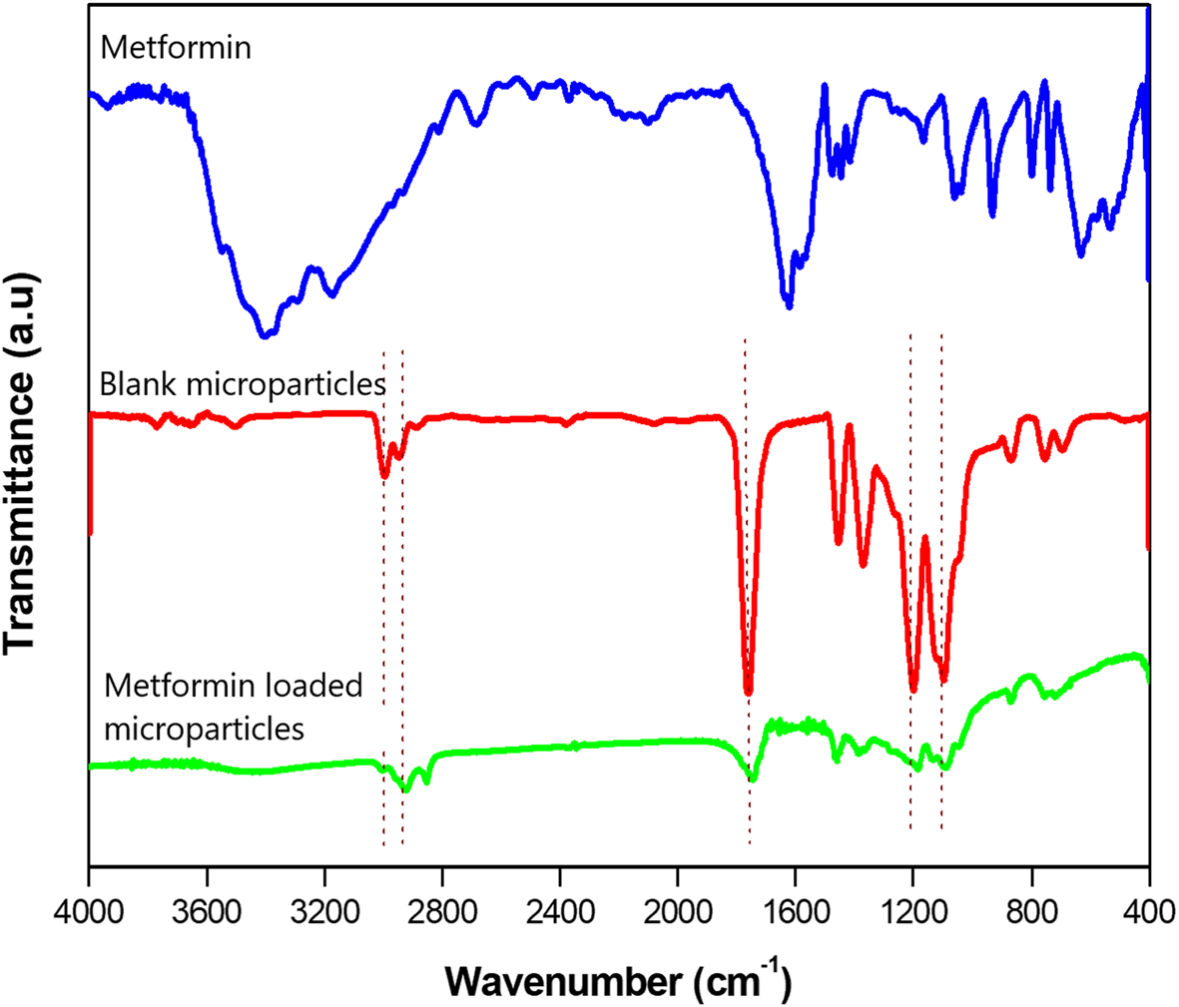
X-ray diffraction (XRD) spectra for pure metformin, blank microparticles and metformin-loaded microparticles scanned between 5–80° at 5θ. Pure metformin (blue curve) displayed sharp, high-intensity peaks typical of the crystalline drug. The XRD curve of blank PLA microparticles (red curve) shows two very intense peaks characteristic of its semi-crystalline structure. On the other hand, microencapsulation of metformin into the PLA microparticles (green curve) suppressed the sharp peaks of the drug, indicating its amorphous nature in the microparticles

FTIR spectroscopy was used to investigate the encapsulation process of the metformin-loaded microparticles. The FTIR spectra of pure metformin, blank PLA microparticles and metformin-loaded microparticles are shown in Fig. 2. The FTIR spectrum of pure metformin (Fig. 2a) shows characteristic peaks at 3390, 1633 and 1045 cm^−1^ that correspond to primary N–H stretching, C = N stretching, and C-N stretching, respectively. For the blank microparticles (Fig. 2), four characteristic peaks were observed at 1764, 2993, 2943 and 1087 cm^−1^, which were attributed to the C = O stretching of the ester group and asymmetric and symmetric vibrations of the CH_3_ group, -CH_3 (asym)_ and CH_3 (sym)_, and the C-O ester bond, respectively. As shown in the microparticle spectrum (Fig. 2), the intensity of the C = O peak from the ester groups of PLA at 1764 cm^−1^ after metformin loading was reduced, which is due to hydrogen bond formation between the carbonyl of ester of PLA and hydrogens of the amine groups of metformin (Fig. 1). It has been reported that elongation of the C = O band to a C-O–H band after hydrogen bond formation results in a shift of the absorption band to a lower wavenumber. These results confirm that metformin was successfully encapsulated in the PLA microparticles.

**Fig. 3:**
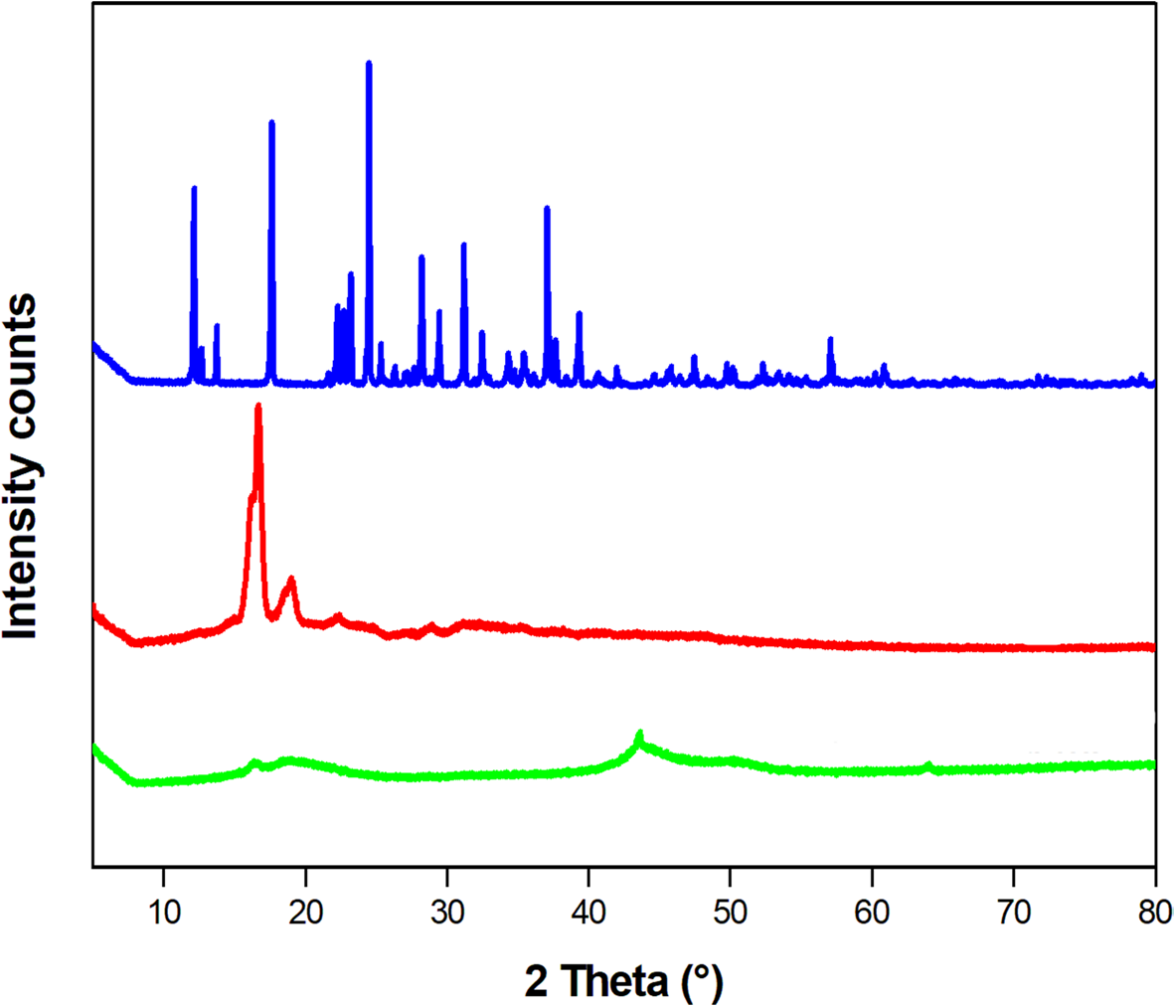
Differential scanning calorimetry (DSC) spectra of pure metformin, blank microparticles and metformin-loaded microparticles at a heating rate of 10 °C min^−1^. The melting peak of metformin is observed at approximately 232 °C (blue curve), and the melting peak of the blank PLA microparticles is observed at 178 °C (red curve). The absence of the characteristic metformin peak and the shift in the PLA peak in the spectrum of the metformin-loaded PLA microparticles (green curve) are significant for determining metformin dispersion inside the PLA microparticles

Figure 2 illustrates the X-ray diffraction (PXRD) diffractograms of the pure metformin, blank microparticles and metformin-loaded microparticles. The PXRD diffractogram of metformin revealed distinct peaks at 2θ: 12.17°, 17.62°, 24.47°, 28.2°, 31.17°, and 37.07°; these sharp peaks confirmed the highly crystalline nature of metformin. The blank microparticles showed peaks at 2θ: 16.6° and 18.92°, indicating the semi-crystalline nature of PLA [17]. The PXRD diffractogram of the microparticles showed no peaks. As observed in Fig. 3, the peaks from both PLA and metformin disappeared compared to the blank microparticles and pure metformin. This may be attributed to the incorporation of metformin into the PLA matrix leading to a change in the crystallinity of PLA and metformin. These results showed good entrapment of metformin in PLA and the corresponding interactions, which aligns with the FTIR analysis.

### Differential scanning calorimetry (DSC)

In addition to FTIR and XRD, DSC analysis was performed to obtain additional information about the physical state of metformin in PLA microparticles and the intermolecular interactions between the drug and polymer. The DSC curves of the samples were recorded during the first heating process (Fig. 3). The metformin DSC curve exhibited a very sharp endothermic peak at ~ 232 °C, which indicates its crystalline nature. The DSC curve of the blank PLA (unloaded) microparticles showed a broad peak at ~ 178 °C, and this peak shifted to 168 °C in the metformin-loaded PLA microparticles. No peak from metformin in the loaded microparticles was observed. Water-soluble metformin interacts with PLA via electrostatic forces between the NH3^+^ and COO^–^ groups; consequently, changes in the thermal behaviours of both metformin and the PLA polymer occur [21]. This result indicates that metformin is dispersed or dissolved in the polymer matrix during the microencapsulation process.

### Pharmacokinetic analysis

**Fig. 4:**
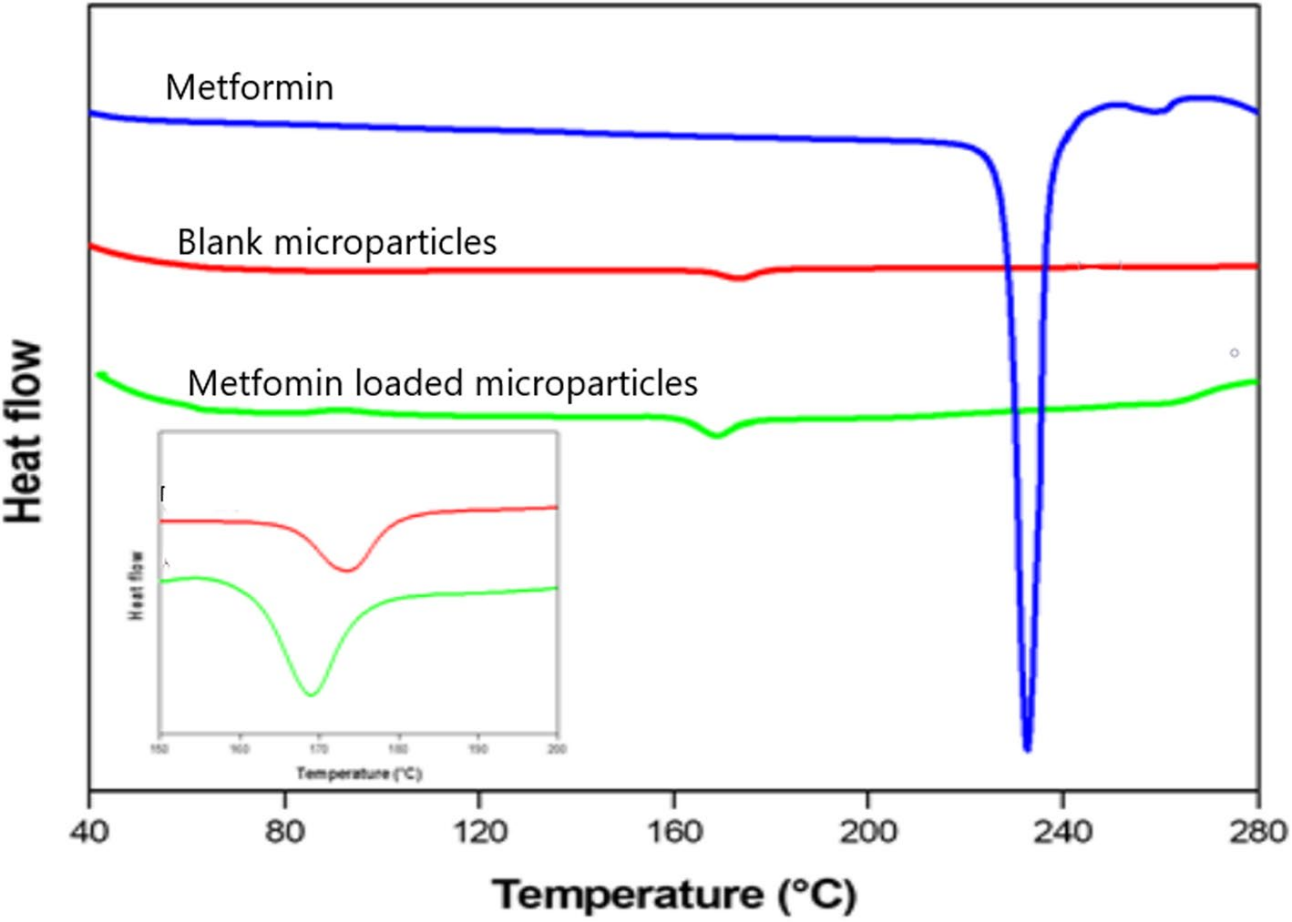
Mean (*n* = 5) plasma concentration–time profiles (semi-logarithmic plot) of metformin after single-dose administration via S_IV,_ S_PO_ and S_PLA_ in rabbits. The dose of the drug administered was 5 mg/kg (± SD) (*p* < 0.05)

An in vivo study was conducted to assess the pharmacokinetics of the metformin-loaded PLA microparticles after three routes of administration: metformin intravenous solution (S_IV_), metformin oral solution (S_PO_) and oral PLA microparticles (S_PLA_). No adverse effects were observed in any of the animals. The mean plasma concentration–time profiles after a single administration at the same dose (5 mg/kg) to rabbits in the S_IV,_ S_PO_ and S_PLA_ groups are illustrated in Fig. 4. The quantification of metformin in plasma after 24 h was determined by HPLC.

After S_PO_ administration, the drug was gradually absorbed and reached maximum absorption at a T_max_ of 110 min with a higher C_max_ of 3.19 ± 0.16 µg/mL followed by a decrease to ~ 1 µg/mL within 300 min. In contrast, the metformin concentration in plasma was maintained at low levels over ~ 600 min after S_PLA_ administration, showing a lower C_max_ (1.36 ± 0.05 µg/mL) and higher T_max_ (138 min) than that after S_PO_ administration. This result may be attributed to the delayed release of metformin from the S_PLA_ microparticles.

The pharmacokinetic parameters derived from the plasma concentration–time data for the different routes are summarized in Table 1. The results indicated that the mean residence time (MRT) was prolonged with S_PLA_ (369.08 min) compared to the MRTs of S_IV_ and S_PO_ (156.52 min, 254.59 min, respectively). The longer MRT enables the maintenance of the concentration of metformin for a prolonged period in rabbit plasma.

**Table 1:** Pharmacokinetic parameters of metformin S_PLA_, S_PO_ and S_IV_ from healthy rabbits after single oral administration of 5 mg/kg body weight to rabbits. Results are presented as the means ± SD (*n* = 5)

**Parameter**	**IV solution**	**Oral solution**	**Oral microparticles**
**C**_**max**_** (µg/mL)**	-	3.19 ± 0.16	1.36 ± 0.05
**T**_**max**_** (min)**	-	110.5 ± 2.37	138.94 ± 4.73
**T**_**1/2**_** (min)**	139.61 ± 4.39	153.78 ± 4.19	223.30 ± 21.30
**AUC**_**0-inf**_** (µg/mL/min)**	2440.77 ± 68.71	882.85 ± 23.90	617.88 ± 12.92
**AUC**_**0-last**_** (µg/mL/min)**	2271.93 ± 64.68	693.36 ± 21.93	512.89 ± 9.68
**AUC**_**360 min**_** (µg/mL/min)**	2271.93 ± 64.68	693.36 ± 21.93	340.56 ± 9.68
**% AUC**_**last-inf**_** (µg/mL/min)**	6.37 ± 0.48	15.08 ± 0.96	16.2 ± 1.23
**AUMC**_**0-∞**_** (µg/mL/min)**	370,257.81 ± 8667.42	176,590.56 ± 6288.57	220,874.35 ± 7235.63
**V**_**Z**_** (mL/kg)**	380.81 ± 22.44	881.45 ± 52.16	2494 ± 212.37
**V**_**SS**_** (mL/kg)**	315.55 ± 16.64	1445.82 ± 52.24	2912.23 ± 199.58
**Cl (mL/min/kg)**	0.830 ± 30.1	0.850 ± 23.57	0.330 ± 20.16
**MRT (min)**	117.73 ± 1.88	165.1 ± 1.53	255.53 ± 1.9
**MAT (min)**	-	63.66 ± 2.13	208.28 ± 31.86
**F (%)**	-	30.74 ± 1.56	22.59 ± 0.69
**F (%)**_**360 min**_	-	-	15.51 ± 1.53

*C*_*max*_ Peak or maximum drug concentration following extravascular administration, *T*_*max*_ Time to reach peak or maximum drug concentration following extravascular administration, *T½* Elimination half-life associated with the terminal (log-linear) elimination phase, *AUC*_*0-inf*_ Area under the concentration–time curve from zero to infinity, *AUC*_*0-last*_ Area under the concentration–time curve from zero to the last observed concentration after S_PO_ administration, *% AUC*_*last-inf*_ Percentage of the AUC_0-inf_ that corresponds from the last administration to infinity, the area under the concentration–time curve from zero to the last observed concentration, *AUC*_*360 min*_ Area under the concentration–time curve from zero to 360 min, *V*_*z*_ Apparent volume of distribution calculated by the area method, *V*_*ss*_ Volume of distribution at steady state, *Cl* Plasma clearance, *MRT* Mean residence time, *MAT* Mean absorption time, *F (%)* Fraction of the administered dose systemically available (bioavailability), *F (%)*_*360 min*_ Bioavailability calculated with AUC_360 min_ as the last point for the last point of the oral solution administration

Additionally, the half-life (t_1/2_) of metformin S_PLA_ (223.30 ± 21.30 min) was longer than the half-lives obtained from the S_PO_ (153.78 ± 4.19 min) and S_IV_ (139.61 ± 4.39 min) routes. This result indicates that incorporation/encapsulation of metformin into the PLA microparticles resulted in a longer half-life than the pure oral solution and may be suitable for sustained-release use.

The AUC_0-last_ after S_PLA_ was 512.89 ± 9.68 µg/mL/min, the AUC_0-360 min_ after S_PLA_ was 512.89 ± 9.68 µg/mL/min while the AUC_0-last_ of the pure metformin solution was 693.36 ± 21.93 µg/mL/min and 2271.93 ± 64.68 µg/mL/min after S_PO_ and S_IV_ administration, respectively. The oral bioavailability of S_PLA_ was calculated to be 22.59 ± 0.69%. The significantly lower AUC_0-inf,_ AUC_0-360,_ and AUC_0-last_ values of S_PLA_ indicate low release of metformin from the developed microparticles compared to the oral solution. In the case of S_PLA_, the decrease in AUC_0-inf_ and the low C_max_ value could be explained by a further decrease in the diffusion of metformin from the PLA microparticles, which limits the release of the drug.

It was also observed that the values of the other pharmacokinetic parameters, such as V_z_, V_ss_, Cl and MAT, of metformin S_PLA_ were higher than those observed for the pure metformin solution after S_PO_ and S_IV_ administration (Table 1).

## Discussion

The oral route is by far the preferred route of drug administration for many dosage forms. However, many anti-diabetic drugs suffer from low bioavailability following oral administration, especially those in BCS class III, such as metformin, due to its high solubility and low permeability [22]. Therefore, microencapsulation into hydrophobic polymer microparticle systems is an effective strategy to achieve sustained release over time and try to enhance drug bioavailability for both human and veterinary medicinal use.

In a previous study, we showed the successful preparation of poly (lactic acid) microparticle-loaded metformin using the double emulsion (W/O/W) solvent evaporation method. In this method, the effects of the microencapsulation parameters, such as the amount of metformin, pH of the external aqueous phase, PVA concentration, stirring rate on the encapsulation efficiency, microparticle size and zeta potential, were evaluated and optimized using response surface methodology by Box–Behnken design [20]. In the present study, microparticles were prepared according to the optimal value of each experimental factor [20]. After microparticle characterization, the results showed the potential of the metformin-loaded poly (lactic acid) microparticles to sustain in vivo release in rabbits under standard feeding conditions.

Metformin-loaded PLA microparticles, under the conditions reported previously, exhibited a high encapsulation efficiency (76 ± 2.72%) and spherical shape without a porous surface. From the FTIR spectra, some variations in the absorption bands were observed before and after loading metformin into the microparticles. These changes could be a result of the physicochemical interactions between the microparticles and metformin when forming the metformin-loaded microparticles during the process of microencapsulation [23]. Solid-state characteristics are important to control drug release. A large amount of energy is required in the crystalline state to separate the molecules, leading to low aqueous solubility and consequently low physiological bioavailability, while the amorphous state needs less energy to separate the molecules; thus, the drug solubility and bioavailability are superior [14]. The SEM, XRD and DSC results indicated that metformin was dissolved in the microparticles via intermolecular interactions between the drug and polymer, and the drug was encapsulated in the microparticles in a non-crystalline state. The crystallinity of metformin and PLA disappeared after microencapsulation because of the fast diffusion of dichloromethane to the outer phase during the evaporation step, thus inhibiting the formation of a crystalline structure [21].

Microencapsulation of metformin in the PLA microparticles caused a change in the pharmacokinetics of the drug in the rabbit model used in this study. The C_max_, T_max_ and bioavailability (F) values reflect the in vivo absorption rate of metformin. In our study, the metformin concentration in plasma was maintained at a low level for 24 h after S_PLA_ administration, demonstrating the slow release of metformin, which may provide an efficient therapeutic concentration for a prolonged period of time. The peak plasma concentration (C_max_ = 1.36 ± 0.05 µg/mL) for metformin after S_PLA_ administration was 2.34-fold lower than the peak plasma concentration via S_PO_. In addition, the time needed to reach the maximum plasma concentration (T_max_) was significantly delayed in the S_PLA_ group, which may be due to the low absorption of metformin in the cells of the gastrointestinal tract. This type of behaviour is expected for PLA microparticles and has been found for extended-release formulations. The mean values of bioavailability (F) obtained from the S_PO_ and S_PLA_ groups were 30.74% and 22.59%, respectively. These values are similar to the bioavailability of metformin in diabetic rabbits (36.73%) [24], rats (34.1%) [25] and dogs (31%) [5] and lower than that in cats (48%) [7] and humans (50–60%) [26]. However, the bioavailability values found in the S_PO_ and S_PLA_ groups were significantly higher than that in horses (3.9%) [4]. It is important to note that in rabbits, the bioavailability in this study was calculated according to AUC_0-inf_, and AUC_0-last_ and AUC_0-360 min_ were used to avoid possible bias, as AUC_0-inf_ only reached 300 min (Fig. 4). The F_360min_ value of the S_PLA_ formulation (15.51%) is promising, showing the characteristic sustained-release pattern found in extended-release formulations. However, it is important to note that the F value for the S_PLA_ formulation is significantly lower than that of the S_PO_ solution. It is also important to note the higher V_z_ and V_ss_ values in the S_PLA_ group with respect to the S_PO_ and S_IV_ groups and the reduction in Cl after S_PLA_ with respect to S_PO_ and S_IV_, which could be due to the progressive uptake of metformin by the functional transporters present in the basolateral membrane of the gut as a result of its sustained release after S_PLA_ administration [26].

**Fig. 5:**
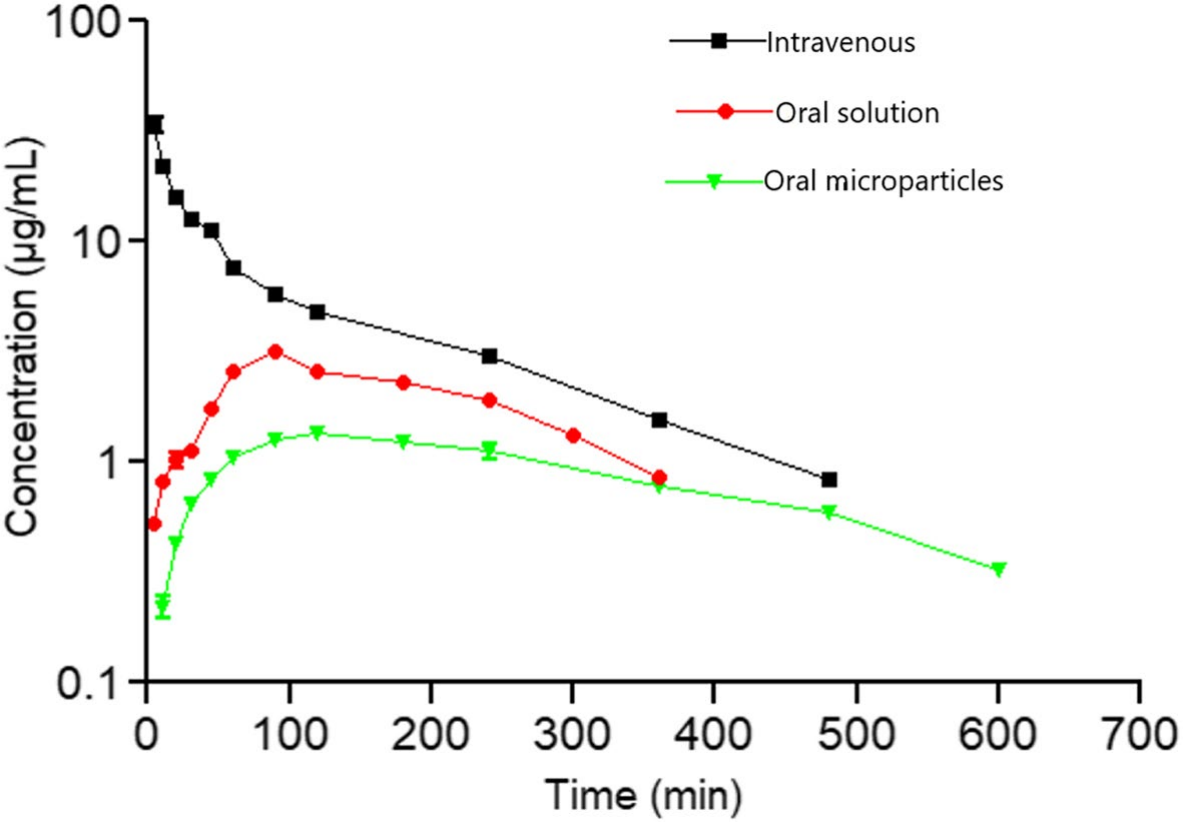
Fourier transform infrared **(**FTIR) spectra of pure metformin, blank microparticles and metformin-loaded microparticles. Metformin characteristic peaks at 1663 cm^−1^ and 3390 cm^−1^ (blue spectrum) were not observed in the metformin-loaded microparticle spectrum (green spectrum), indicating encapsulation of the drug. The characteristic PLA C = O peak at 1764 cm^−1^ (red spectrum) remained at practically the same position in the metformin-loaded microparticle spectrum (green spectrum), with the changes in its absorption indicating that metformin was successfully entrapped in the PLA microparticles

Particle size and size distribution can influence the in vivo dissolution rate as well as the oral bioavailability of a drug. As shown from the SEM image (Fig. 5), the obtained PLA microparticles had a large particle size that led to slower degradation of the polymer matrix. As a result, delayed drug diffusion through the PLA microparticles occurred, which may explain the lower C_max_ and AUC_0-inf_ values of metformin S_PLA_ compared to S_PO_ and consequently the lower bioavailability [27].

The nature of the drugs to be encapsulated is an important factor that influences drug release from the polymer matrix [28]. The experimental FTIR and DSC findings suggested the presence of an interaction between the protonated metformin and the carboxylic end groups of PLA, which affects drug release and leads to slow release over time. Our results are consistent with those obtained by Proikakis et al. [28]. In addition, the amorphous state of metformin in the microparticles could enhance the release of the drug; however, this was not the case in our study. This result could be due to the slow degradation of the PLA polymer [29]. Therefore, a promising extended-release PLA microparticle formulation of the highly water-soluble drug metformin was successfully designed.

However, some limitations of this study should be noted. First, the lack of previous pharmacokinetics studies of metformin in rabbits makes it difficult to choose the optimal dose to be tested. There has been only one PK study of metformin in rabbits [24], which used a very high dose (30 mg/kg) for other purposes. Additionally, extrapolation from human doses is not the most recommended practice; therefore, the low dose of 5 mg/kg was selected due to the widely known gastrointestinal issues that metformin can produce. This limitation could cause bias in the interpretation of the results and could influence the calculated F value, as F is calculated using the dose-dependent parameter AUC. In future studies, consideration of the use of higher doses would be necessary. Second, we did not perform an in vitro study of the microparticle formulation. Although an in vitro test was successfully conducted in a previous publication, in this case, this sort of study could complement the information from the in vivo study, and although the lack of in vitro results did not have an impact on the in vivo study results, this should be considered in future studies. Some of these limitations may have had an influence on some of the results obtained that were unexpected, such as the lower bioavailability of the microparticle formulation with respect to the oral solution.

## Conclusions

Metformin-loaded PLA microparticles showed optimal and beneficial properties in terms of their physicochemical characteristics, making them suitable for use in an in vivo pharmacokinetic study. The pharmacokinetic parameters from the in vivo study showed a shorter T_max_, longer MRT and half-life, and decreased C_max_ of metformin from the microparticles, showing the prolonged/sustained release expected for metformin. However, the unexpected decrease in bioavailability with respect to S_PO_ should be evaluated with the microparticles and dose designs in future works, especially before being tested in other animal species in veterinary medicine.

## Methods

### Materials

Metformin HCl had a Mw of 165.6 g/mol. Poly (lactic acid) (PLA) was purchased from Evonik Industries AG (Germany). Poly (vinyl alcohol) (PVA; 87–89% hydrolysed, average Mw = 30,000–70,000) was provided by Sigma Aldrich (Barcelona, Spain). High-performance liquid chromatography (HPLC)-grade acetonitrile, methanol and water were purchased from Sigma Aldrich (Barcelona, Spain), and sodium dodecyl sulfate (SDS) ultrapure (288.38 g/mol) and ammonium acetate (77.08 g/mol) were purchased from Panreac (Barcelona, Spain). All other reagents were of analytical grade.

### Animals

Fifteen healthy female rabbits were obtained from the Faculty of Veterinary of the University of Murcia (Murcia, Spain). The rabbits were housed in cages in laboratory animal rooms of the Laboratory Of Pharmacy, Pharmacology and Therapeutics of the Veterinary Faculty. The animals were kept on a photoperiod of 12 h light/12 h dark. They received a standard laboratory chow diet (Nanta, Madrid, Spain) and tap water. All experimental protocols were carried out in accordance with the requirements of the applicable national legislation and approved by the Bioethics Committee of the University of Murcia (CEE-628/2020).

### Preparation of metformin HCl-microparticles

Metformin HCl (Sigma-Aldrich, Madrid, Spain)-loaded PLA microparticles were prepared by the W/O/W solvent evaporation method as described previously. In this method, four process variables, the amount of metformin, the pH of the external aqueous phase, the PVA concentration and the stirring rate, were optimized, and the optimal conditions to obtain a high EE were used in this study to prepare the microparticles. The in vitro test of the optimized microparticles showed sustained release of metformin in simulated gastric and intestinal fluids [20]. Briefly, 1 mL of aqueous internal phase (50 mg of metformin, Span® 80 at 5% v/v) was emulsified for 5 min in 10 mL of methylene chloride (containing 200 mg of PLA) in an ultrasonic bath (Branson 5510, BioBlock Scientific, Spain) at 135 W output. This primary emulsion was gradually added to 40 mL of a 1.5% PVA aqueous solution and homogenized for 30 min at 700 rpm to create the W/O/W emulsion. Solvent evaporation was achieved at room temperature and atmospheric pressure under 400 rpm agitation (Heidolph Hei-Tec D91126, Germany). Microparticles were obtained after centrifugation of the colloidal suspension for 30 min at 3000 × g (Centrifuge 5702, Eppendorf Ag, Germany). Drug-free microparticles (placebo microparticles) were prepared according to the same procedure. The microparticles were frozen and lyophilized at -50.0 ± 0.5 °C and 0.13 mbar for 24 h (Alpha1-4 LD Plus Freeze Dryer).

### Determination of the encapsulation efficiency (EE)

The amount of metformin entrapped within the polymeric microparticles was determined by measuring the amount of non-encapsulated metformin in the external aqueous solution, recovered after centrifugation of the microparticles, by high-performance liquid chromatography (JASCO series HPLC) equipped with a Kromasil C-18 column (250 mm × 4.6 mm, 5 µm, Análisis Vínicos S.L., Spain) thermostatted at 45 °C. The mobile phase was acetonitrile:potassium dihydrogen phosphate buffer (0.02 M, pH = 6.8, (50:50, v/v)), delivered at a flow rate of 1.0 mL/min. The wavelength was fixed at 236 nm. The assay was repeated using different samples from independent preparations. The drug loading capacity (DLC) and encapsulation efficiency (EE) were calculated using the following equations:

1 $$DLC=\frac{weight\;of\;drug\;in\;microparticles}{weight\;of\;microparticles}\times100$$

2 $$EE=\frac{weight\;of\;drug\;in\;microparticles}{weight\;of\;fed\;drug\;initially}\times100$$

### Microparticle characterization

#### Scanning electron microscopy (SEM)

The morphology of the metformin-loaded microparticles was investigated by scanning electron microscopy (SEM) (Zeiss Evo 50, Germany) at a working distance of 9.5 mm and accelerating voltage of 8 kV. The microparticles were prepared by fixation on carbon adhesive tape and sputter-coating with a 10 nm Pd/Au layer under vacuum (EVO/LS 15). The microparticles were examined for their shape, size and surface characteristics.

#### Fourier transforms infrared (FTIR) spectroscopy

To examine the drug-polymer interactions, the FTIR spectra of pure metformin, blank PLA microparticles and metformin-loaded microparticles were obtained with an IR Affinity-1 CE spectrophotometer (Shimadzu, Japan). One milligram of each sample was finely mixed with 100 mg of purified potassium bromide and pressed in a mechanical die press to form a pellet (90 N, 5 min). These pellets were scanned, and spectra were recorded from 400 to 4000 cm^−1^ at a resolution of 4 cm^−1^.

#### X-Ray powder diffraction (XRPD)

Pure metformin, blank PLA microparticles and metformin-loaded microparticles were investigated by wide angle X-ray diffraction (XRPD) using an X-ray diffractometer (X Per-Pro, PANalytical, Netherland). All samples were scanned at 40 kV and 30 mA using Cu-Kα radiation (λ = 1.54059 Å) in the range of 2θ from 5 to 80 °C with a scanning speed of 5° min^−1^.

#### Differential scanning calorimetry (DSC)

The thermal properties of the samples were evaluated by differential scanning calorimetry (DSC-131, Setaram, France). DSC thermograms of pure metformin, blank PLA microparticles and metformin-loaded microparticles were obtained using SETSOFT software. The samples (8–12 mg) were weighed and sealed into aluminium pans; an empty sealed pan was used as a reference. DSC curves were obtained at a heating rate of 10 °C from 30–300 °C.

### Pharmacokinetics study

Fifteen healthy New Zealand white rabbits (*n* = 15) were selected for the pharmacokinetics study. The rabbits were randomly divided into 3 groups (*n* = 5) (the Microsoft® Excel® 2016 MSO 16.0 random numbers table generator was used to generate pseudo-random numbers from the uniform distribution Mersenne Twister algorithm) and received a single oral dose of metformin microparticle suspension (Group A), pure metformin solution (Group B) by gastric intubation using an oral feeding needle, or S_IV_ administration via injection into the marginal ear vein (Group C). The microparticles and pure metformin were suspended/dissolved in saline before each administration at a dose of 5 mg/kg body weight. At predetermined time points after drug administration (10, 20, 30, 45, 60, 90, 120 min and 4, 6, 8, 10, 24, 34 and 48 h) blood samples were taken. The blood sampling design should ensure that no measurable drug concentrations are missed, especially in the case of the PLA formulation; therefore, a longer than expected sample-taking design is needed. Blood samples were extracted (0.5 mL) from the marginal ear vein of the rabbits with a heparinized syringe and immediately centrifuged (3000 × *g*, 10 min) to separate the plasma, which was frozen at -50 °C until further analysis. Blood samples (1 mL) were collected by inserting a 20-gauge needle into the marginal ear vein and allowing the blood drip into the heparinized syringe. No anaesthesia or pain management was needed. After the experiment, the rabbits were returned to the farm after being checked for good health.

#### Plasma analysis

To quantify the amount of drug in rabbit plasma, a validated HPLC validated [17] modified by Carceles-Rodriguez et al. [3] was used. First, the samples were thawed. Then, acetonitrile (200 µL) was added to each plasma sample (200 µL), followed by vortexing for 15 s, shaking in an ultrasonic bath for 5 min to allow complete mixing, and centrifugation at 12,000 rpm for 10 min to extract the metformin. Afterwards, 200 µL of the supernatant was mixed with the HPLC mobile phase (ratio of 1:3), and the sample was transferred to an HPLC vial and analysed. A standard calibration curve was generated with the metformin from plasma. The established linear range was 100–10,000 µg/L (*r* > 0.99).

#### Pharmacokinetics analysis

The plasma metformin time-concentration data were analysed with PKanalix software version 2020-R1 using a non-compartmental approach (Antony, France: Lixoft SAS, 2020). The area under the concentration–time curve (AUC_**0-∞**_) and the area under the first moment (AUMC_**0-∞**_) were calculated using the linear trapezoidal rule with extrapolation to infinity, and the area under the concentration–time curve until the last point (AUC_**0-last**_) was constructed using data to the last measured time point. The mean residence time was calculated as MRT = AUMC_**0-last**_/AUC_**0-last**_. The systemic clearance was calculated as Cl = dose/AUC_**0-last**_. The apparent volume of distribution (area method) and apparent volume of distribution at steady state were calculated as V_z_ = Dose/(AUC_**0-last**_·λ_z_) and V_ss_ = (dose·AUMC_**0-last**_)/AUC_**0-last**_^2^, respectively. The mean absorption time was calculated as MAT = MRT _(extravascular routes)_ − MRT_IV_, and the bioavailability F (%) was determined by the method of corresponding areas.

#### Statistical analysis

Descriptive statistical parameters, such as the mean, standard deviation (SD) and coefficient of variation (CV), were calculated. Harmonic means were calculated for the elimination and absorption half-lives. The *Kruskal–Wallis test* was used to check for normal distribution of the parameters and concentration ranges between animals. The *Wilcoxon rank sum and Student’s t tests* were used to test the parameters for significant differences according to the route of administration. The statistical software used was SPSS Version 19.0 (SPSS Statistic Program, Chicago, USA). Values of *P* < 0.05 were considered significant.

## Data Availability

The datasets used during the current study are available from the corresponding author on reasonable request.

## Abbreviations

PLA: Poly (lactic acid)
S_IV_: Pure metformin solution
S_PO_: Pure metformin oral solution
S_PLA_: Metformin-loaded microparticle oral solution
T_max_: Time to reach maximum concentration
C_max_: Maximum concentration
MRT: Mean residence time
BCS: Biopharmaceutics Classification System
CDDSs: Polymeric-controlled drug delivery systems
SEM: Scanning electron microscopy
FTIR: Fourier transform infrared spectroscopy
XRPD: X-ray powder diffraction
DSC: Differential scanning calorimetry
HPLC: High-performance liquid chromatography
EE: Encapsulation efficiency
AUC_0-∞_: Area under the concentration–time curve
SD: Standard deviation
CV: Coefficient of variation
